# First Isolation and Characterization of a Group C Banna Virus (BAV) from *Anopheles sinensis* Mosquitoes in Hubei, China

**DOI:** 10.3390/v10100555

**Published:** 2018-10-11

**Authors:** Han Xia, Hong Liu, Lu Zhao, Evans Atoni, Yujuan Wang, Zhiming Yuan

**Affiliations:** 1 Key Laboratory of Special Pathogens and Biosafety, Wuhan Institute of Virology, Chinese Academy of Sciences, Wuhan 430071, China; hanxia@wh.iov.cn (H.X.); zhaolu20181010@outlook.com (L.Z.); atonet@live.com (E.A.); 2 School of Life Sciences, Shandong University of Technology, Zibo 255000, China; liuhongseminar@126.com; 3 University of Chinese Academy of Sciences, Beijing 10049, China; 4 Henan Province OriginBio Biotechnology Co., Ltd., Zhengzhou 450000, China; wangyj1988@outlook.com

**Keywords:** Banna virus, novel genotype, group C, Hubei

## Abstract

Banna virus (BAV) is considered to be an emerging human pathogen that is transmitted by blood-sucking insects. BAV was isolated from various species of mosquitoes, midges, and livestock. It is widely distributed geographically, since it was identified in China, Vietnam, and Indonesia. Previously reported evolution studies of BAV indicated that BAV can be divided into two groups, including isolates from China and Vietnam clustered in group A, and Indonesian isolates in group B. In this study, we report the isolation of a new strain of BAV named HB14-71-01 from *Anopheles sinensis* mosquitoes from Hubei, China. An in vitro comparison study of the HB14-71-01 isolate and the group A BAV revealed differences based on observed cytopathic effect, plaque size, and viral growth rates. Additionally, the phylogenetic analysis indicated that the Hubei isolate belongs to a novel genotype of BAV and emerged nearly 105 years ago (95% highest posterior density (HPD): 35–434), unlike the two previously reported genotypes A and B. Our findings extend the knowledge about the genomic diversity and potential vectors/hosts of BAVs and will improve understanding of the relationships between genetic variation and pathogenicity.

## 1. Introduction

Banna virus (BAV) is a virus grouped under the genus *Seadornavirus* within the *Reoviridae* family [1]. BAV is considered to be an emerging pathogen which is suspected to cause human infections with fever and viral encephalitis. BAV was first isolated from the cerebrospinal fluid and sera of human patients with encephalitis in Xishuangbanna, Yunnan province, China in 1987 [2]. Subsequently, BAVs were isolated from mosquitoes, ticks, midges, cattle, and pigs in China, Vietnam, and Indonesia [3,4,5].

BAV is a double-stranded RNA (dsRNA) virus composed of 12 segments and is approximately 19.5 kb in genome length. Segments 1–4 and 8–10 encode for the structural proteins of BAV, while segments 7 and 12 encode for the protein kinase and the dsRNA-binding protein, respectively. The remaining segments encode for nonstructural proteins with unknown function [6,7].

Previous research studies on BAV found that BAV strains cluster significantly according to their geographical distribution. Phylogenetic analysis based on the complete coding sequence of segments 9 or 12 indicate that BAV is divided into two groups: A and B [3,8,9,10]. Isolates from China and Vietnam are included in group A, while group B comprises Indonesian strains that were isolated in the regions below 15° north (N) latitude. Additionally, group A can be divided into two subgroups; the group A1 strains were isolated in the regions beyond 30° N latitude, and the group A2 strains were isolated between 15° N and 30° N latitude.

In China, Banna virus has a wide geographic distribution since it was isolated from various regions including Gansu, Shanxi, Inner Mongolia, Liaoning, Beijing, Hubei, and Yunnan. All these previous isolates from China belong to group A [10,11,12,13,14,15]. Here, we report a new BAV strain, HB14-71-01, which was isolated from *Anopheles sinensis* mosquitoes collected in Yichang, Hubei, China in 2014. This new isolate has phenotypic and genomic characteristics diverging from the BAV group A viruses and is differently clustered, forming a new genotype—group C.

## 2. Materials and Methods

### 2.1. Mosquito Collection, Treatment, and PCR Screening

Adult mosquitoes were caught in Yichang, Hubei province, China (N 31°03′32″, E 111°38′39″) in 2014. Trapped mosquitoes were killed by flash-freezing in liquid nitrogen, and they were then morphologically classified into four species: *Anopheles sinensis*, *Armigeres subalbatus*, *Culex quinquefasciatus*, and *Culex tritaeniorhynchus*. The samples were grouped into pools of 50 and kept frozen at −80 °C until further analysis.

The pooled mosquitoes were homogenized, and the extracts were clarified using centrifugation as described previously [16]. The homogenate samples were passed through sterile 0.22-μm microfilters (Millipore, Billerica, MA, USA) to remove bacteria and other debris. The filtered supernatant samples were used for BAV screening and subsequent virus isolation.

RNA was extracted using a QIAamp viral RNA extraction kit (Qiagen, Hilden, Germany) following the manufacturer’s instructions. To screen for BAVs, one-step reverse-transcription polymerase chain reaction (one-step RT-PCR) was conducted using a Prime ScriptTM one-step RT-PCR kit Ver.2 (Takara, Dalian, China) and two primer sets, 12-B2-S: 5’–CAGAGTATAAATCAATCGCCCAAG–3’ and 12-B2-R: 5’–GTTCTAAATTGGATACGGC GTGC–3’ [17]. The reaction mixture (total volume of 25 µL) contained 12.5 µL of 2 × One-Step Buffer, 1.0 µL of Prime Script one-step enzyme mix, 1.0 µL of each primer (10 µM), 7.5 µL of H_2_O, and 2.0 µL of RNA sample; thermal cycling was performed at 50 °C for 30 min and 94 °C for 2 min, followed by 35 cycles at 94 °C for 30 s, 56 °C for 30 s, and 72 °C for 30 s. PCR products were visualized in a 1.5% agarose gel with ethidium bromide.

### 2.2. Virus Isolation, Purification, and Electron Microscopy

Supernatants of positive pools were inoculated onto monolayers of *Aedes albopictus* C6/36 cells. Cytopathic effects (CPEs) were observed daily, followed by RT-PCR detection of BAV.

The culture supernatant was harvested after at least two blind passages, and clarified to remove cellular debris by centrifugation at 5000× *g* for 30 min at 4 °C; it was then centrifuged with 20% sucrose cushion at the bottom of the tube (28,000× *g*, 2 h, 4 °C) using an SW32 Ti rotor (Beckman, Brea, CA, USA). The supernatant was discarded, and the virus pellet was resuspended in 200 μL of phosphate-buffered saline (PBS). The resuspended pellet was ultracentrifuged in a continuous sucrose gradient (20–70% *w*/*v*) in an SW41 Ti rotor at 39,000 rpm for 2 h; sucrose was removed from the purified fractions by centrifugation with 200 μL of PBS at 40,000 rpm for 1 h at 4 °C. The supernatant was discarded, and the pellet was resuspended in PBS and stored at −80 °C. Electron microscopy was performed using the negative contrast method. The pure virus particles were added to a Formvar carbon-coated copper grid for 10 min and negatively stained for 2–3 min with 2% phosphotungstic acid (PTA) at pH adjusted to 6.8 with 1 M KOH; the samples were examined using a Hitachi U8010 electron microscope (Hitachi, Tokyo, Japan).

### 2.3. Plaque Assay and Growth Kinetics

BAV strain YN15-126-01 was isolated from the *Culex tritaeniorhynchus* mosquito pool from Yunnan, 2015; this species has a highly similar (>95%) genome sequence with the other reported BAVs isolated from Yunnan which belong to group A. To compare the basic viral characteristics between HB14-71-01 and YN15-126-01, a plaque assay and a growth kinetics assay were performed simultaneously. Prepared aliquots of 10-fold serial dilutions of the virus in Rosewell Park Memorial Institute (RPMI) medium were inoculated onto the C6/36 cell monolayers in 24-well plates for 2 h. The cells were then covered with an overlay medium including 1.5% methylcellulose and incubated at 28 °C for 4–6 days to allow for plaque development. Afterward, the infected cells were stained with 2% crystal violet in 30% methanol for 5 min at room temperature. The plaques were manually counted and measured.

For the growth kinetics assay, the C6/36 cells in a T75 flask were infected with the BAV viruses YN15-126-01 or HB14-71-01 (multiplicity of infection (MOI) = 0.001), and 2 mL of supernatant was collected from the flask, before 2 mL of fresh medium was re-added every day until all cells were either floating or dead. The virus titers of the collected samples were detected using the plaque assay, and the experiments were repeated three times.

### 2.4. Genome Sequencing and Polymerase Chain Reaction (PCR) Confirmation

The viral RNA was extracted from the passaged virus and sent to Shanghai Biotechnology Corporation (Illumina Miseq System) for sequencing. The viral sequences were edited and assembled using AbySS v2.0.2 [18] and SOAPdenovo [19]; they were further verified with PCR using multiple primers. The full genome sequences (VP1 to VP12) were submitted to GenBank and the accession numbers are listed in Table 1.

### 2.5. Bioinformatics Analyses

The nucleotide sequence identity of the complete coding sequences (CDSs) of a newly isolated Hubei BAV and five other strains (SC0143, 02VN018b, 02VN078b, JKT-6423, QTM104536, and BAV_Ch available in GenBank as of June, 2018) were analyzed using BioEdit v7.0.5 [20].

The 12th segment genes of 30 BAV strains and representative members of the *Seadornavirus* genus (Banna-like virus, Liao ning virus, Mangshi virus, and Kadipiro virus) were used for the analysis, and the background information is listed in Table 1 [1,21,22,23]. Alignment of the BAVs with the representative members of the *Seadornavirus* genus was conducted using the ClustalW module, and phylogenetic analyses were conducted with the maximum-likelihood method with 1000 bootstrap replicates using MEGA v7.0.21 [24]. Bayesian time-scaled phylogenetic analysis, molecular evolutionary rates, and the most recent common ancestor (MRCA) of the BAVs were co-estimated using the BEAST software package v1.8.4 [25]. The GTR + I + G substitution model was selected by the MrModelTest software [26]. The relaxed clock model with different demographic models was tested, and the best models were selected using a Bayes factor (BF) test with the marginal likelihood values (2lnBF > 2) and 95% highest posterior density (HPD) intervals. The analysis was run through 100,000,000 generations to ensure sufficient mixing. Finally, the maximum clade credibility (MCC) was built using TreeAnnotator with 10% burn-in.

## 3. Results

### 3.1. BAV Detection and the Genome of BAV HB14-71-01

We screened 44 mosquito pools to determine the prevalence of BAV in the field-collected mosquitoes. Six mosquito pools were PCR-positive for BAV, indicating that the prevalence of BAV infection in mosquitoes in Yichang is approximately 13.6%. Only one BAV isolate was successfully obtained from a pool of *Anopheles sinensis.* The isolate was named HB14-71-01.

De novo assembly of the next-generation sequenced data acquired 99.5% coverage of the complete BAV genome. The lengths for segments 1–12 were 3752, 3059, 2449, 2037, 1637, 1656, 1118, 1105, 1070, 957, 830, and 852 bp, respectively. The complete CDS of VP1 to VP12 encoded 1219, 954, 720, 628, 481, 427, 306, 302, 282, 245, and 208 amino acids, respectively.

### 3.2. Viral Plaque and Growth Kinetics for BAV Strain HB14-71-01

**Figure 1:**
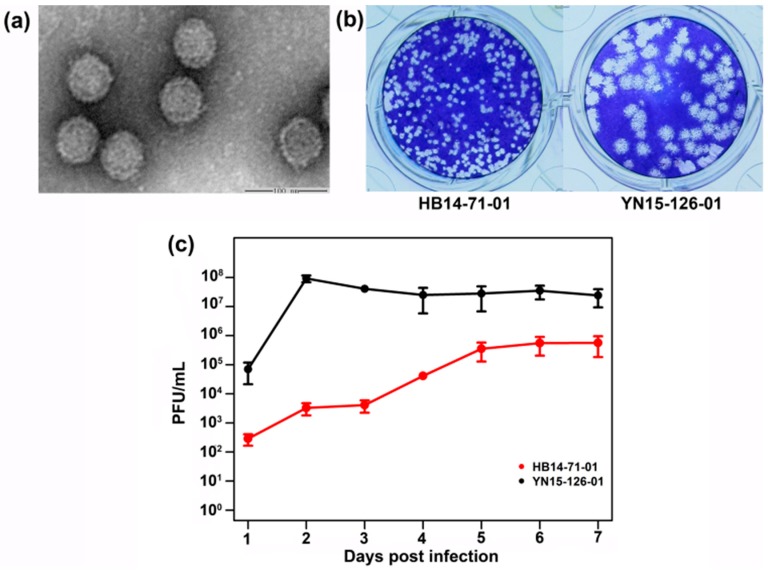
Viral particle, plaque, and growth kinetics for Banna virus (BAV) HB14-71-01. (**a**) Purified viral particles of BAV HB14-71-01 were observed under an electron microscope (EM); (**b**) viral plaques of BAV HB14-71-01 and YN15-126-01 in the C6/36 cells; (**c**) growth kinetics of BAV HB14-71-01 and YN15-126-01 in C6/36 cells.

A negatively stained image showed that the BAV strain HB14-71-01 virus particles are non-enveloped with a diameter of approximately 60 nm (Figure 1a). The BAV HB14-71-01 strain was clearly different from the group A BAV (YN15-126-01 in Figure 1), including weaker CPEs, smaller-sized plaques, and slower growth rates in the C6/36 cells (Figure 1a–c). Three days after inoculation, CPEs caused by YN15-126-01 were very clearly manifested by increasing cell gaps and cell shedding. The lesion frequency reached 50%; however, the cells infected with HB14-71-01 showed only small changes (Supplementary Materials, Figure S1). Two days post inoculation, the viral titer reached a maximum at 10^8^ plaque-forming units (PFU)/mL for YN15-126-01; however, the highest titer for HB14-71-01 was achieved in six days post infection at 10^5^ PFU/mL (Figure 1c). Seven days post inoculation, most part of the cells were floating or dead in the flasks infected by HB14-71-01 or YN15-126-01.

### 3.3. Gene Sequence Comparison of BAVs with Full Genomes

The nucleotide sequence identity analysis of all 12 segments was conducted among HB14-71-01 and other 5 BAV isolates with full genome information (Table 2). The results indicate that HB14-71-01 is closely related to the recently reported QTM104536 with an identity of over 80% for all genes except the VP10 gene. Excluding the VP11 gene, the identity of the other 11 segments of HB14-71-01 compared with that of SC043, 02VN078b, 02VN018b, or JKT-6423 was less than 80%.

### 3.4. Phylogenetic Analysis among BAVs and Other Members of the Seadornavirus Genus

**Figure 2:**
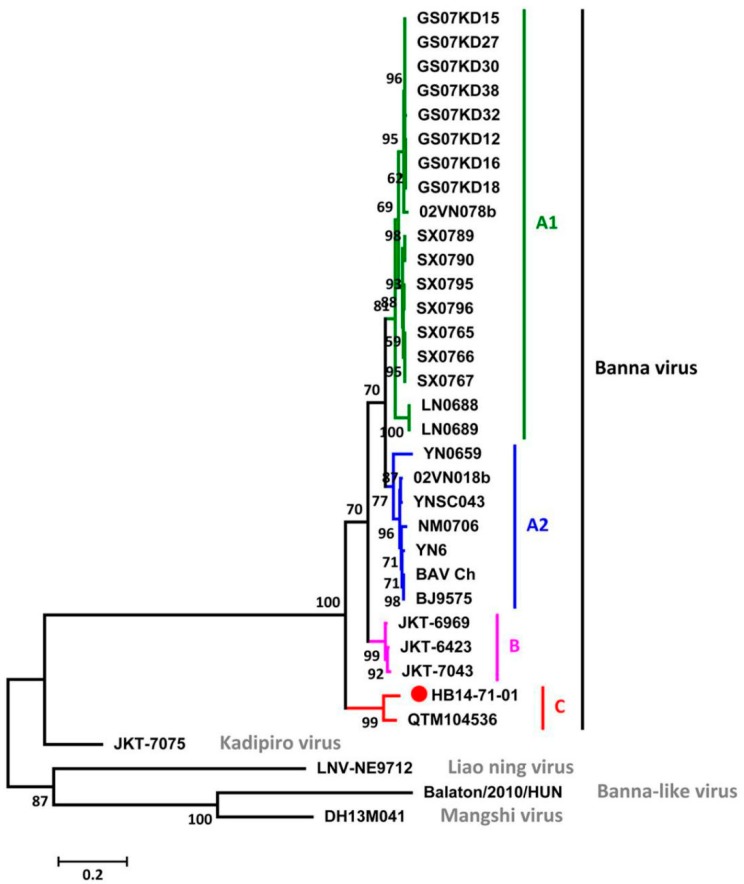
Phylogenetic analysis of the complete coding region of BAVs and other members of the *Seadornavirus* genus based on the 12th segment, which encodes for the outer coat protein. BAV groups A1, A2, B, and C are labeled in green, blue, violet, and red, respectively. Other representative seadornaviruses were labeled in grey.

The phylogenetic relationships among BAV and other representative strains such as Banna-like virus (BALV), Liao ning virus (LNV), Mangshi virus, and Kadipiro virus (KDV) of the *Seadornavirus* genus were analyzed based on segment 12. The maximum-likelihood tree reveals that HB14-07-01 was distinct from BALV, KDV, LNV or Mangshi virus, and it clustered together with other BAVs (Figure 2). Additionally, HB14-07-01 and QTM104536 formed an independent group within the BAVs. These results suggest that BAVs can be genotypically classified into three groups—A, B, and C—based on segment 12, and that the new BAV isolate HB14-71-01 belongs to group C.

### 3.5. Time-Scaled Evolutionary Analysis of BAVs

**Figure 3:**
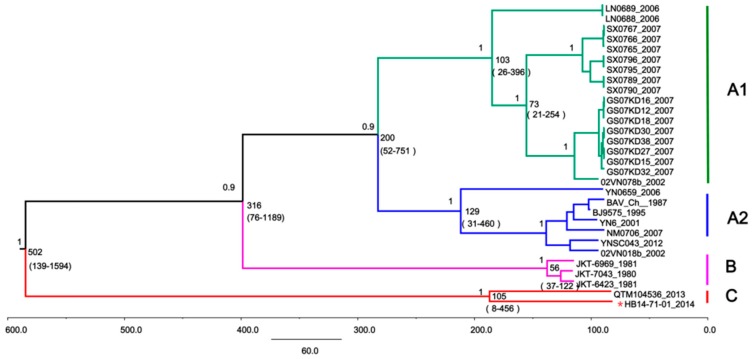
Time-scale evolutionary analysis of BAVs based on segment 12. The tree identifies three distinct lineages: genotypes A (including subgroups A1 (green) and A2 (blue)), B (violet), and C (red). The estimated most recent common ancestors (MRCAs) of these lineages (with 95% highest posterior density (HPD) values in parentheses) are presented. The newly isolated Hubei strain HB14-71-01 is indicated with a red star.

Based on the segment-12 genome sequences, a maximum clade credibility tree was generated for BAVs (Figure 3). The MRCA of BAV existed approximately 502 years ago (95% HPD: 109–1837) and gradually evolved into three major evolutionary populations and groups A, B, and C. The MRCA of group A, which consists of subgroups A1 and A2, was estimated to have emerged 213 years ago. Group B, which includes strains mainly isolated from Indonesia, appeared approximately 56.9 years ago (95% HPD: 37–119). Interestingly, the newly isolated Hubei strain together with the QTM104536 strain formed an independent evolutionary branch that emerged nearly 105 years ago (95% HPD: 35–434), thus indicating that it is a new BAV lineage. The mean nucleotide substitution rate for the entire BAV population was estimated to be at 2.467 × 10^−4^ substitutions per site per year (s/s/y) (95% HPD: 1.093 × 10^−4^ to 5.628 × 10^−2^).

## 4. Discussion

Previously reported BAV genotypes (group A and group B) are significantly clustered according to the geographical point of virus isolation. BAV group A is further divided into subgroup A1, comprising strains from north China, and subgroup A2, comprising strains from south China and Vietnam [3,8,9,10]. BAV group B represents strains obtained from Indonesia. Based on the findings of our study, we report a novel BAV strain from Hubei that expands the present genotype classification into groups A, B, and C, based on the maximum-likelihood method and Bayesian Markov chain Monte Carlo (MCMC) analysis using the MEGA and BEAST software, respectively. The newly clustered group C consists of two BAV strains obtained from a mosquito and Odonata from central China; they exhibit high genetic diversity versus the previously reported groups A and B, thus forming a distinct genetic group. This finding extends current knowledge about genomic diversity and potential vectors/hosts of BAVs and further supports our previous findings that BAVs are rapidly evolving viruses that have great transmissibility and host adaptability, and that are prevalent in mainland China [8,9].

Additionally, Gao et al. previously reported the isolation of four BAV strains from *Cx. tritaeniorhynchus* and *An. sinensis* in Hubei, China, in 2009 and phylogenetic analysis of their 12th segment (published in Chinese; sequence information not available in GenBank), indicating that these isolates belong to subgroup A2 alongside other BAV isolates from Yunnan, Inner Mongolia, Beijing, and Vietnam. However, in this study, a new Hubei BAV isolate from *An. sinensis* obtained in 2014 does not cluster in subgroup A2, but rather, clusters in group C, thus expanding the diversity of natural BAVs.

HB14-71-01 and QTM104536 were clustered together to form group C and were reported in a mosquito collected in 2014 and in Odonata collected in 2013, respectively. However, the sequencing information for QTM104536 was obtained from the metagenomic analysis, and no virus isolate and phenotypic information was reported for this strain [27]. Quite a number of the arboviruses do not induce CPEs in insect cells; however, BAV could produce CPEs in C6/36 cells. The current study reports biological information, including replication ability within the C6/36 and mammalian cells, CPEs, viral plaques, and growth rates for HB14-71-01. Similar to other reported BAVs, the HB14-71-01 strain cannot replicate in mammalian cell lines such as BHK-21 and Vero. The cytotoxicity of this new isolate of group C was weaker than that of a representative strain of group A in the C6/36 cells. Due to the lack of a representative BAV isolate from group B, we do not know if the viral characteristics of the group C virus are similar or not to the group B virus. It would be interesting to do a comparison among all three genotypes of BAV in the future to investigate the factors that may influence the viral characteristics.

It should be noted that only a few BAV strains have their full genome information in the National Center for Biotechnology Information (NCBI) GenBank; hence, whole-sequence analysis is currently limited only to the analysis of segments 9 or 12. When additional full BAV genomes are revealed in the future, evolutionary analysis based on other segments will substantially improve understanding of these viruses.

## Figures and Tables

**Table 1:** Background information for viruses used in the study.

Virus	Isolate	Source	Location	Accession Number		Time of Collection
				1st to 11th segments	12th segment	
Banna virus	02VN018b	*Culex Annulus*	Vietnam: Quang Binh	EU265683; EU265684; EU265685; EU265686; EU265687; EU265688; EU265689; EU265690; EU265691; EU265692; EU265693	EU265694	2002
	02VN078b	*Cx. tritaeniorhynchus*	Vietnam: Ha Tay	EU265695; EU265696; EU265697; EU265698; EU265699; EU265700; EU265701; EU265702; EU265703; EU265704	EU265705	2002
	BAV_CH	Human	China: Yunnan	AF168005; AF134526; AY549307; AY549308; AY549309; AF168006; AF052035; AF052034; AF052033; AF052032; AF052031	AF052030	1987
	BJ9575	Unidentified mosquito	China: Beijing		AY568289	1995
	GS07-KD12	*Anopheles sinensis*	China: Gansu		GQ331954	2007
	GS07-KD15	*Cx. tritaeniorhynchus*	China: Gansu		GQ331955	2007
	GS07-KD16	*Cx. pipiens pallens*	China: Gansu		GQ331956	2007
	GS07-KD18	*An. sinensis*	China: Gansu		GQ331957	2007
	GS07-KD27	*Cx. tritaeniorhyn*	China: Gansu		GQ331958	2007
	GS07-KD30	*Cx. pipiens pallens*	China: Gansu		GQ331960	2007
	GS07-KD32	*Cx. pipiens pallens*	China: Gansu		GQ331961	2007
	GS07-KD38	*Cx. pipiens pallens*	China: Gansu		GQ331962	2007
	JKT-6423	*Cx. pseudovishnui*	Indonesia: Java		NC_004198	1981
	JKT-6969	*Aedes vagus*	Indonesia: Java		AF052008	1981
	JKT-7043	*Cx. pipiens pallens*	Indonesia: Java		AF052024	1980
	LN0688	*An. sinensis*	China: Liaoning		FJ217990	2006
	LN0689	*An. sinensis*	China: Liaoning		FJ217991	2006
	NM0706	*Cx. modestus*	China: Inner Mongolia		CQ331973	2007
	SX0765	*Cx. pipiens pallens*	China: Shanxi		CQ331963	2007
	SX0766	*Cx. pipiens pallens*	China: Shanxi		CQ331964	2007
	SX0767	*Ae. vexans*	China: Shanxi		CQ331965	2007
	SX0789	*Ae. dorsalis*	China: Shanxi		CQ331967	2007
	SX0790	*Ae. vexans*	China: Shanxi		CQ331968	2007
	SX0795	*Cx. pipiens pallens*	China: Shanxi		CQ331971	2007
	SX0796	*Cx. pipiens pallens*	China: Shanxi		CQ331972	2007
	YN0659	*An. sinensis*	China: Yunnan		FJ161965	2006
	YN6	Unidentified mosquito	China: Yunnan		AY568290	2001
	YNSC043	*Culicoides* sp.	China: Yunnan	KC954611; KC954612; KC954613; KC954614; KC954615; KC954616; KC954617; KC954618; KC954619; KC954620; KC954621	KC954622	2004
	HB14-71-01	*An. sinensis*	China: Hubei	MH521264; MH521265; MH521266; MH521267; MH521268; MH521269; MH521270; MH521271; MH521272; MH521273; MH521274	MH521275	2014
	QTM104536 ^1^	*Odonata*	China: Hubei	KX884638; KX884639; KX884640; KX884641; KX884642; KX884643; KX884644; KX884645; KX884646; KX884647; KX884649	KX884648	2013
Banna-like virus	Balaton/2010/HUN ^2^	*Cyprinus carpio*	Hungary: Veszpre		JX947850	2010
Mangshi virus	DH13M041	*Cx. tritaeniorhynchus*	China: Yunnan		KR349198	2013
Kadipiro virus	JKT-7075	*Cx. fuscocephalus*	Indonesia: Java		NC_004199	1981
Liao ning virus	LNV-NE9712	*Ae. dorsalis*	China: Liaoning		NC_007747	1997

^1,2^ QTM104536 and Balaton/2010/HUN were identified using viral metagenomic analysis.

**Table 2:** Nucleotide sequence identity matrix for Banna viruses (BAVs). NS—nonstructural.

Banna Virus Isolates	VP1 (Pol/Core)	VP2 (T2/Core)	VP3 (Cap/Core)	VP4 (Outer Coat)	VP5 (NS)	VP6-(NS)	VP7-(NS)	VP8 (T13-Core)	VP9 (Outer Coat)	VP10 (Core)	VP11-(NS)	VP12-(NS)
	HB14-71-01											
YNSC043	0.755	0.748	0.717	0.766	0.715	0.766	0.739	0.790	0.652	0.696	0.829	0.780
02VN078b	0.757	0.744	0.704	0.760	0.697	0.761	0.744	0.772	0.639	0.698	0.815	0.778
02VN018b	0.762	0.740	0.702	0.762	0.713	0.747	0.728	0.790	0.645	0.696	0.830	0.784
JKT-6423	0.766	0.733	0.700	0.765	0.701	0.762	0.743	0.781	0.650	0.678	0.831	0.790
QTM104536	0.881	0.879	0.904	0.834	0.895	0.875	0.908	0.879	0.848	0.798	0.907	0.925
BAV_Ch	0.757	0.721	0.661	0.672	0.645	0.679	0.736	0.788	0.652	0.695	0.827	0.775

## Supplementary Materials

The supplementary material is available online at http://www.mdpi.com/1999-4915/10/10/555/s1.
