# Supplementary figures and images for: First Isolation and Characterization of a Group C Banna Virus (BAV) from Anopheles sinensis Mosquitoes in Hubei, China
Source: Viruses. 2018 Oct 11;10(10):555. doi: 10.3390/v10100555 (PMC6213526; doi:10.3390/v10100555)

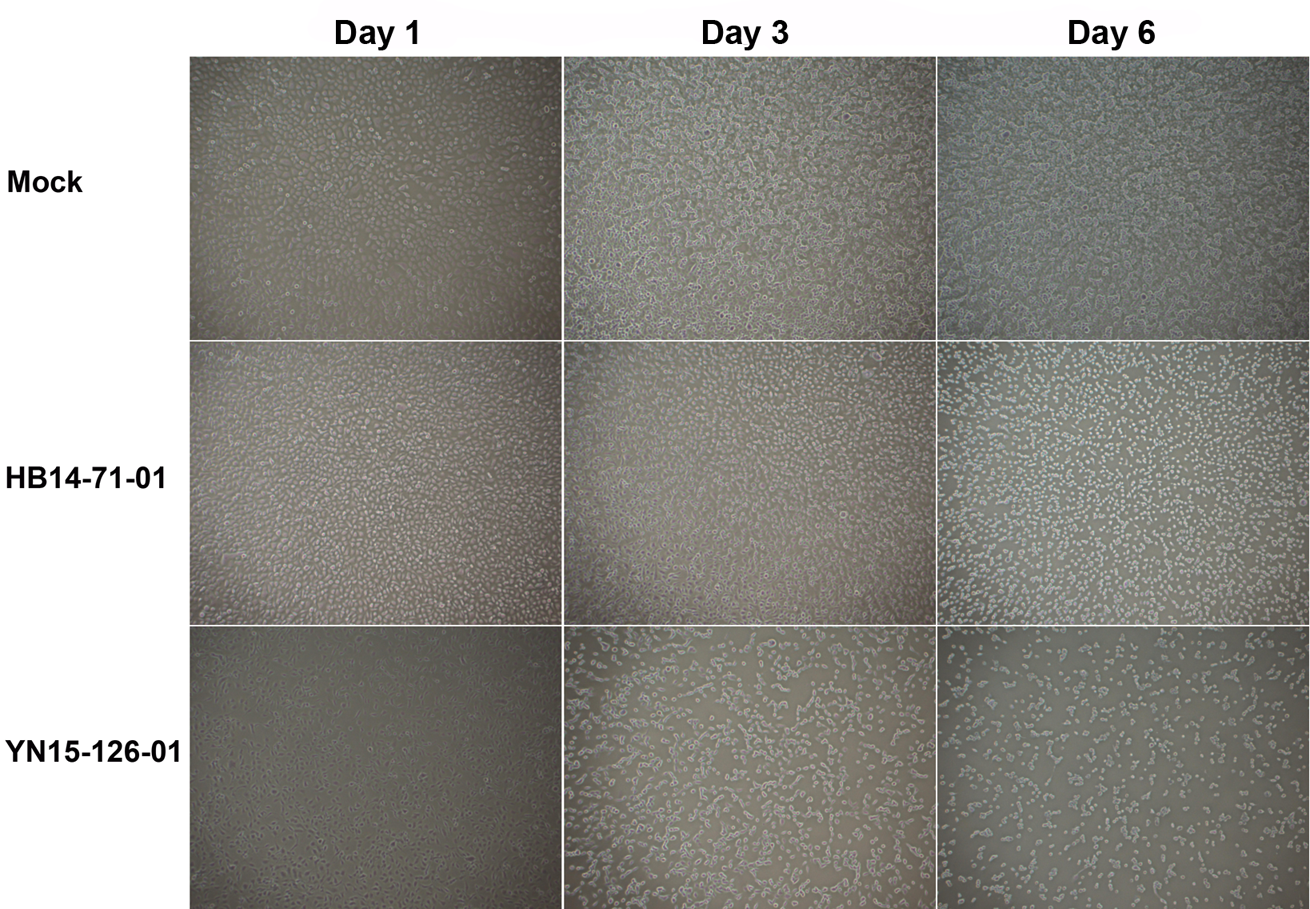

Supplement: Supplementary file 1 [file viruses-10-00555-s001.jpg]
